# *In silico* study of the potential of curcumin and its derivatives for increasing wild-type p53 expression and improving the function of p53 mutant R273H

**DOI:** 10.14202/vetworld.2025.715-730

**Published:** 2025-03-31

**Authors:** Sarah Ika Nainggolan, Rajuddin Rajuddin, Reno Keumalazia Kamarlis, Muhammad Hambal, Frengki Frengki

**Affiliations:** 1 Graduate School of Mathematics and Applied Sciences, Universitas Syiah Kuala, Banda Aceh 23111, Indonesia; 2 Department of Obstetrics and Gynecology, Division of Gynecological Endocrinology, Faculty of Medicine, Syiah Kuala University, Banda Aceh, Indonesia; 3 Department of Parasitology, Faculty of Medicine Veterinary, Syiah Kuala University, Banda Aceh, 243111, Indonesia; 4 Department of Pharmacology, Faculty of Medicine Veterinary, Syiah Kuala University, Banda Aceh, Indonesia

**Keywords:** curcumin derivatives, *in silico* analysis, molecular docking, molecular dynamics, p53 enhancer, p53 mutant R273H

## Abstract

**Background and Aim:**

p53 is a critical tumor suppressor protein responsible for regulating the cell cycle and inducing apoptosis. Mutations in the *p53* gene, particularly in the DNA-binding domain, are frequently associated with various cancers due to the loss of transcriptional activity. Curcumin and its derivatives have demonstrated potential as p53 enhancers and reactivators of mutant p53. This study employs *in silico* methods to evaluate the potential of curcumin derivatives to enhance wild-type p53 expression and restore the function of the p53 mutant R273H.

**Materials and Methods:**

Curcumin and 20 derivatives were selected from PubChem for computational analysis. Their potential as p53 enhancers was assessed using Quantitative Structure-Activity Relationship (QSAR) analysis. Molecular docking was conducted to determine their binding affinities with wild-type and mutant p53 proteins, followed by molecular dynamics (MD) simulations to evaluate ligand-receptor stability. Pharmacokinetics and toxicity assessments were performed using predictive computational models to evaluate their drug-like properties.

**Results:**

QSAR analysis identified hexahydrocurcumin (probable activity [Pa]: 0.837) and tetrahydrocurcumin (Pa: 0.752) as the most potent p53 enhancers. Molecular docking revealed strong binding affinities for curcumin derivatives at key p53 binding residues, particularly through hydrogen bonds with His 273 of the R273H mutant. MD simulations demonstrated that curcumin, bisdemethoxycurcumin, and monodemethylcurcumin stabilized p53 mutant R273H, closely mimicking the structural stability of wild-type p53. Pharmacokinetic analysis indicated favorable absorption, distribution, metabolism, and excretion profiles for most derivatives, with low toxicity predicted for the majority.

**Conclusion:**

Curcumin and its derivatives exhibit dual functions as p53 enhancers and reactivators of the p53 mutant R273H. Hexahydrocurcumin and tetrahydrocurcumin emerged as promising compounds with strong bioactivity and favorable pharmacokinetic properties, suggesting their potential as anticancer agents. Further *in vitro* and *in vivo* studies are necessary to validate these findings and explore their therapeutic applications.

## INTRODUCTION

One of the main causes of death worldwide and a factor preventing most countries from reaching their goal of a longer life expectancy is cancer [1]. Cancer is the leading cause of death in the productive age group in 177 of 183 countries. At least 1 in 6 deaths (16.8%) occurring worldwide and 1 in 4 deaths (22.8%) are reported due to non-communicable diseases come from cancer sufferers [2]. Half of the people undergoing advanced cancer therapy worldwide often succumb to the disease due to treatment-related effects. p53 disorders are identified in almost all types of cancer, with a very high percentage. Qi *et al*. [3] stated that p53 mutations occur in many types of tumors, such as colon and gastric tumors, each identified as 60%, lung 70%, esophageal 60%, brain 40%, and breast 20%. Well-known and widely used chemotherapy drugs, such as cisplatin, paclitaxel, 5-fluorouracil, and doxorubicin, are toxic to cancer cells and healthy cells and have side effects that weaken the patient’s body [4]. Curcumin is one of the many natural compounds that have been reported to have a multifunctional mechanism of anticancer activity, not only in resistant cancer types but also in cancer cell types with multiresistant xenografts.

Previous studies by Liu *et al*. [5], Shaikh *et al*. [6], and Fuloria *et al*. [7] have reported that curcumin administered with chemotherapy has a synergistic effect, reducing cell resistance to drugs and the dose of biologically active drugs. Several studies by Liu *et al*. [5], Shaikh 71 *et al*. [6], Fuloria *et al*. [7], Sharifi-Rad *et al*. [8] have shown that turmeric has few side effects and is very safe for long-term use [5–8]. In cancer therapy, curcumin has also been shown to inhibit Akt activation and decrease the expression of cyclooxygenase-2; 5-lipooxygenase; vascular endothelial growth factor; phosphorylated signal transducers and activators of transcription 3; and matrix metalloproteinase-9, while increasing the expression and function of p53 in triggering apoptosis, all of which are closely related to tumorigenesis [9, 10]. p53 is known as “the guardian of the genome” [11] because it can control the cell cycle and trigger apoptosis and senescence. Mutations in the *p53* gene occur in almost all types of human and animal cancers [12, 13]. In humans, it is often observed in tumors such as breast carcinoma, prostate cancer, adenocarcinoma, osteosarcoma, leukemia, and lymphoma [14]. In animals such as dogs, p53 mutations have also been identified in various tumors such as thyroid carcinoma, mammary tumors, osteosarcoma, oral papilloma, lymphoma, and hemangiosarcoma [15–18]. A study of curcumin targeting the *p53* gene and protein was conducted by Lee and Kweon [15], who tested the effects of curcumin on cell viability in three ovarian cancer cell lines, including SKOV3 (p53 null-type), MDAH2774 (p53 mutant), and PA1 (p53 wild-type). Lee and Kweon [15] revealed a decrease in cell viability and apoptosis in ovarian cancer cells but not in normal cells. In addition, inhibition of sarco/endoplasmic reticulum calcium ATPase activity by curcumin stimulated an increase in cytosolic Ca^2+^ concentrations and resulted in apoptosis regardless of p53 status. These data suggest that curcumin is an effective specific anticancer agent for ovarian cancer with minimal toxicity to normal cells [19]. Curcumin has also been reported to increase the expression of cell cycle control genes and pro-apoptosis (p53, p73, PPARy, and Bax) while suppressing genes involved in cell cycle stimulation and anti-apoptosis (MDM2, MAPK, Akt, and Bcl-2) [20]. MDM2 as a p53 ubiquitination complex is one of the ten main targets of curcumin based on a networking analysis and is closely related to the PI3K/Akt signaling pathway, ErbB pathway, hypoxia-inducible factor-1 pathway, and forkhead box O (FOXO) pathway, which are involved in the process of cancer cell proliferation, angiogenesis, invasion, metastasis, and chemotherapy resistance [21]. Talib *et al*. [20] also described p53 as an important target of curcumin, which is directly involved in the antiproliferative mechanism while triggering apoptosis in cancer cells. Curcumin has even been shown *in vitro* to be able to restore the function of p53 mutant Y220C in pancreatic adenocarcinoma BxPC-3 cell lines and p53 mutant R273H in HT-29 (American Type Culture Collection [ATCC]) cell lines [22, 23]. Mutations in p53 are often found in the DNA-binding domain (DBD) region in the form of point mutations in the amino acids Arg 175, Arg 249, Arg 282, Arg 248, and Arg 273, which cause loss of contact between p53 and DNA so that p53 transcription activity fails [12, 18]. Interestingly, derivatives and metabolites of curcumin, such as dihydrocurcumin, tetrahydrocurcumin, hexahydrocurcumin, and octahydrocurcumin, which are the results of reductase enzyme metabolism, are also reported to provide pharmacological effects similar to those of curcumin above [24]. Dihydrocurcumin *in vitro* has been reported to improve nonalcoholic fatty liver disease liver cancer cell models [25]. The antitumor effects of hexahydrocurcumin and octahydrocurcumin are also demonstrated through the activation of apoptosis factors and inhibition of nuclear factor kappa B transcription factor function *in vivo* [24, 26, 27]. Tetrahydrocurcumin is reported to have antioxidant, anti-neurodegeneration, anti-aging, and anti-cancer effects [28]. Modification of the chemical structure of curcumin is not only intended to increase its bioactivity but is also expected to improve the profile of its physicochemical and pharmacokinetic properties so that its therapeutic effect is more optimal [29]. Here, some curcumin derivatives have been reported to have several advantages over curcumin. The presence of methyl group substitutions, such as dimethyl curcumin compounds, has been reported to increase the therapeutic effect of anticancer drugs against prostate and breast cancers [30, 31]. Metal complexes formed by β-diketones such as in vanadium and gallium compounds, increase the cytotoxic effect while improving DNA binding [32, 33], hydrogenated diketone moiety, such as in tetrahydrocurcumin compounds, triggers increased mitochondrial membrane permeability during lymphoma therapy [34], and glycosylated curcumin derivatives have better solubility and are also reported to have the ability to form chelates so that anticancer potential increases [35]. Tetrahydrocurcumin has also been reported to suppress the viability of H22 cancer cells better than curcumin through p53 activation and MDM2 inhibition [36]. Eldar *et al*. [37] reported a p53 mutant R273H model with a change in the 273^rd^ amino acid (arginine to histidine), resulting in the loss of contact interaction between the 273^rd^ amino acid and the DBD. This condition is believed to cause the failure of p53 to stop the cell cycle and trigger apoptosis or senescence. Malami *et al*. [22] confirmed that curcumin can bridge the contact between p53 mutant R273H and DNA through hydrogen bonds facilitated by curcumin through molecular docking. Perdrix *et al*. [38] explained the important role of small molecules such as PRIMA-1 and APR-246 in repairing p53 mutant (R273H) dysfunction. Ahmadi *et al*. [39] reported that the APR-24 compound has reached phase I/II clinical trials for hematological malignancies, prostate cancer, and lymphomas. Curcumin has also been reported to suppress the role of mdm2 while restoring the function of p53 mutant Y220C in pancreatic adenocarcinomas BxPC-3 cell lines and p53 mutant R273H in HT-29 (ATCC) cell lines [22, 23].

Despite the extensive research on curcumin’s anticancer properties, its precise molecular mechanism in enhancing wild-type p53 expression and restoring the function of mutant p53 remains unclear. While previous studies have demonstrated curcumin’s ability to modulate multiple signaling pathways involved in tumorigenesis, there is a lack of comprehensive in silico evaluations exploring its specific interactions with p53 and its mutant variants. Moreover, the potential of curcumin derivatives in stabilizing mutant p53 proteins, particularly the R273H variant, has not been fully characterized. In addition, the pharmacokinetic and toxicity profiles of curcumin derivatives remain underexplored, limiting their translational potential in drug development. This study aims to bridge these gaps by systematically investigating the dual role of curcumin and its derivatives as p53 enhancers and reactivators of the p53 R273H mutant using computational approaches, including quantitative structure activity relationship (QSAR) analysis, molecular docking, molecular dynamics (MD) simulations, and pharmacokinetic assessments.

This study aims to evaluate the potential of curcumin and its derivatives to enhance wild-type p53 expression and restore the function of the p53 mutant R273H through *in silico* approaches. Specifically, the study seeks to (1) assess the bioactivity of curcumin derivatives as p53 enhancers using QSAR analysis, (2) determine their binding interactions with wild-type and mutant p53 proteins through molecular docking, (3) analyze ligand-receptor stability through MD simulations, and (4) predict their pharmacokinetic and toxicity profiles to assess their drug-like properties.

## MATERIALS AND METHODS

### Ethical approval

This study is a digital experiment and was not conducted on laboratory animals, so ethical approval is not required.

### Study period and location

This study was conducted for 7 months (March to September 2023) at the Pharmacology and Computer Laboratory, Faculty of Veterinary Medicine, Syiah Kuala University.

### Tools and materials

The research materials were curcumin ligand and 20 derivatives, which were copied in Simplified Molecular Input Line Entry System (SMILES) format and receptor targets in 3D format. We downloaded the SMILES structure of the ligand through the web server www.pubchem.org and the 3D structure of the receptor [p53 wild-type (pdb id. 2ahi), p53 mutant (pdb id. 4ibs), and mdm2 (pdb id. 4lwu)] through the web server www.rscb.org. Docking of ligands to proteins was carried out using hardware with the following specifications: Central Processing Unit 8.00 GB Random Access Memory Intel^®^ Core (TM) i5 2.50 GHz with Windows 10 64-Bit Operation System (Acer Inc., New Taipei City, Taiwan). Meanwhile, the softwares used were the MOE application 2019 Ver 01.01 (Molecular Operating Environment) (developed by Chemical Computing Group, Inc. (Montreal, Canada), Statistical Package for the Social Sciences (SPSS) Version 2024 (IBM Corp., NY, USA) and Chimera 1.13.2 and is supported by web servers such as http://www.way2drug.com/PASSOnline/predict.php, https://tox.charite.de/protox3/, and https://biosig.lab.uq.edu.au/pkcsm/.

### Methods

#### Research procedure

The identities of curcumin derivatives were obtained from literature sources such as ScienceDirect, PubChem, Scopus, ProQuest, and Google Scholar.

#### Prediction of the potential of curcumin derivatives for increasing p53 expression using QSAR analysis Way2Drug

The potential of curcumin derivatives in increasing p53 expression was determined using a web server (http://www.way2drug.com/PASSOnline/predict.php) by inputting SMILES data for each tested curcumin derivative downloaded from PubChem. The predicted value of bioactivity is known as a “p53 enhancer.” The p53 enhancer is the ability of p53 to regulate the enhancement of elements responsible for DNA damage [40]. “p53 enhancer” is denoted by a probable activity (Pa) and probable inactivity (Pi) value in the range 0.0–1.0. Curcumin derivatives are declared active as “p53 enhancers” if they have a Pa value is > Pi [41]. In studying the structural part of curcumin derivatives that play the most important role in increasing the effect as a “p53 enhancer,” curcumin and all curcumin derivatives were analyzed for their physicochemical properties statistically through multiple linear regression (MLR) equations using the QSAR tools of MOE software (https://www.chemcomp.com/en/index.htm). The physicochemical properties of curcumin and curcumin derivatives are described through eight descriptors, including lipophilicity parameters, such as logP, logS, and topology polar surface area (tpsa); electronic parameters, such as am1_homo and am1_dipole; and steric parameters, using descriptors, such as mr, glob, and vol.

#### Molecular docking of curcumin and its derivatives as mdm2 receptor inhibitors

Validation of the docking method against the native ligand bound to the mdm2 receptor initiates the ligand-protein docking process to be performed. If the native ligand copy’s variation from the native ligand copy’s Root Means Square Deviation (RMSD) value is ≤2 Å, the docking approach is considered valid; if this condition is achieved, then the pose and geometry of the native ligand become the reference “binding site” for the docking process of other test ligands [42]. The results of the native ligand docking validation were used as a reference for curcumin ligands and their derivatives with the “Placement” settings using “Alpha Triangle” and “Refinement” using “Force Field” and “Rescoring” using “London dG.” The p53 R273H mutant has no native ligand bound to it, so the validation process is not performed; thus, the ligand “binding site” follows the MOE “site finder” selection by default.

Furthermore, ligand and receptor optimization was performed by adding hydrogen atoms, adjusting the partial energy, and conditioning the ligand and receptor energies to a minimum to obtain the most stable binding affinity value. In the “binding site” region, the docking procedure is executed in accordance with all previously verified native ligand docking specifications. Molecular docking data in the form of affinity data (∆Gbinding) and ligand-receptor interaction models visualized using LigPlot MOE and Chimera 1.13.2.

#### MD

The MD of the wild-type p53 protein, p53 mutant R723H, and p53 mutant R723H complex docked with curcumin and its derivatives were simulated using the MOE MD tool at a normal body temperature of 310°K for 2 ns. The MD process was carried out under number - temperature-pressure (Constant Temperature and Pressure) conditions. The heating stage was carried out for 10 pc to increase the system temperature to the equilibrium stage. The time was set in the dynamics box of the run column for 2000 pc. Then, a cooling stage was performed for 10 pc to determine the lowest conformational energy of the molecule. This process is known as annealing. The position, velocity, and acceleration results are stored at 1 pc.

#### Ligand toxicity and pharmacokinetic evaluation

Prediction of the potential toxicity of curcumin and its 20 derivatives using lethal dose 50% (LD_50_) mol/kg parameters and toxicity levels referring to the globally harmonized system (GHS) of classification of labeling of chemicals. This toxicity data were downloaded from the protox_ii website based on the report of Banerjee *et al*. [43]. The pharmacokinetic profile of the ligand, including absorption, was determined using the Human Intestinal Absorption (HIA) value and Caco-2 cell permease. The distribution profile shows the plasma protein binding (PPB) and blood-brain barrier (BBB) values; the metabolism profile shows the status of Cytochrome P450 (CYP) substrates and CYP inhibitors; and the excretion profile shows the total clearance value. The pharmacokinetic data were downloaded from the predicting pharmacokinetic small-molecule(pkCSM) website based on the report of Pires *et al*. [44].

### Statistical analysis

All statistical analyses were performed to validate the predictive models and assess the significance of the observed effects. QSAR data were analyzed using MLR to establish a relationship between physicochemical descriptors, such as lipophilicity, electronic, and steric properties, and p53 enhancer activity. The MLR model was evaluated using metrics such as the coefficient of determination (R²), standard error of estimate, and p-values of individual descriptors.

The molecular docking results were statistically validated using RMSD analysis to confirm the docking accuracy. Ligand-binding affinities (ΔGbinding) were compared using descriptive statistics, and significant binding interactions were identified based on hydrogen bonding and hydrophobic interactions with key residues. MD simulations were analyzed using Root Mean Square Fluctuation and RMSD values to evaluate protein-ligand complex stability. Comparisons were made between wild-type p53, mutant p53 R273H, and ligand-bound complexes.

The QSAR model and docking results were further subjected to statistical tests, including Duncan’s multiple range test to identify significant physicochemical descriptors influencing p53 enhancement and analysis of variance to determine the overall significance of the regression model. Toxicity and pharmacokinetic predictions were summarized as descriptive statistics, with threshold values, such as HIA >70% and LD_50_ >0.5 g/kg, used to classify compounds into activity categories.

All statistical analyses were conducted using SPSS software version 24.0 (IBM Corp., NY, USA) and MOE (2019 version 01.01). A significance level of p < 0.05 was considered statistically significant for all tests. Results are presented as mean ± standard error unless otherwise stated.

## RESULTS AND DISCUSSION

### Structure search of curcumin compounds and their derivatives from reliable literature

A structure search of curcumin compounds and their derivatives was performed using PubChem and several articles that have been reported by previous researchers. The potential as a “p53 enhancer” of curcumin and its 20 derivatives was predicted using the QSAR Way2Drug technique by inputting the “smiles” data of each compound on the effect search button. Table 1 below shows the results of the “smiles” structure and “Pa score” of curcumin and its 20 derivatives as “p53 enhancers.”

**Table 1:** Structure of “smiles” and “Pa score” of curcumin and its 20 derivatives.

Compounds	SMILES	References	Pa score
Curcumin (1)	COC1 = C (C = CC( = C1) C = CC( = O) CC( = O) C = CC2 = CC( = C (C = C2) O) OC) O	PubChem CID 969516	0.671
Bisdemethoxycurcumin (2)	C1 = CC( = CC = C1C = CC( = O) CC( = O) C = CC2 = CC = C (C = C2) O) O	PubChem CID 5315472	0.646
Curcumin diglucoside (3)	COC1 = C (C = CC( = C1) C = CC( = CC( = O) C = CC2 = CC( = C (C = C2) OC3C (C (C (C (O3) CO) O) O) O) OC) O) OC4C (C (C (C (O4) CO) O) O) O	PubChem CID 46173989	0.728
Dimethylcurcumin (4)	COC1 = C (C = C (C = C1) C = CC( = CC( = O) C = CC2 = CC( = C (C = C2) OC) OC) O) OC	PubChem CID 6477182	0.631
Didemethylcurcumin (5)	C1 = CC( = C (C = C1C = CC( = O) CC( = O) C = CC2 = CC( = C (C = C2) O) O) O) O	PubChem CID 5469425	0.653
Dimethoxycurcumin (6)	COC1 = C (C = C (C = C1) C = CC( = O) CC( = O) C = CC2 = CC( = C (C = C2) OC) OC) OC	PubChem CID 9952605	0.591
α-curcumene (7)	CC1 = CC = C (C = C1) C (C) CCC = C (C) C	PubChem CID 92139	0.532
β-curcumene (8)	CC1 = CCC( = CC1) C (C) CCC = C (C) C	PubChem CID 6428461	0.557
Monodemethylcurcumin (9)	COC1 = C (C = CC( = C1) C = CC( = O) CC( = O) C = CC2 = CC( = C (C = C2) O) O) O	PubChem CID 5469426	0.671
Pentagamavunon-1 (10)	CC1 = CC( = CC( = C1O) C) C = C2CCC( = CC3 = CC( = C (C( = C3) C) O) C) C2 = O	PubChem CID 10760152	0.644
Diarylpentadienone (11)	OC1 = CC = CC = C1C( = O)\C = C\C = C\C1 = CC = CC = C1	PubChem CID 9532	0.610
(2-hydroxy-4-[(1E,6E)-7-(4 - hydroxy-3-methoxyphen yl)-3,5-dioxo-1,6-heptadie n-1-yl]-2-methoxyphenyl ester) (12)	COC1 = CC(\C = C\C( = O) CC( = O)\C = C\C2 = CC = C (OC( = O) C3 = C (O) C = CC = C3) C (OC) = C2) = CC = C1O		0.644
Dibenzoylmethane (13)	C1 = CC = C (C = C1) C( = O) CC( = O) C2 = CC = CC = C2	PubChem CID 8433	0.610
Demethoxycurcumin (14)	COC1 = C (C = CC( = C1) C = CC( = O) CC( = O) C = CC2 = CC = C (C = C2) O) O	PubChem CID 5469424	0.693
Dihydrocurcumin (15)	COC1 = C (C = CC( = C1) C = CC( = O) CC( = O) C = CC2 = CC( = C (C = C2) O) OC (O) O) O	PubChem CID 87261199	0.600
Hexahydrocurcumin (16)	COC1 = C (C = CC( = C1) CCC (CC( = O) CCC2 = CC( = C (C = C2) O) OC) O) O	PubChem CID 5318039	0.837
Octahydrocurcumin (17)	COC1 = C (C = CC( = C1) CCC (CC (CCC2 = CC( = C (C = C2) O) OC) O) O) O	PubChem CID 11068834	0.720
Tetrahydrocurcumin (18)	CC( = O) OC1 = C (C = C (C = C1) CCC( = O) CC( = O) CCC2 = CC( = C (C = C2) OC( = O) C) OC) OC	PubChem CID 124072	0.752
Tetrahydrocurcumin glucuronide (19)	COC1 = C (C = CC( = C1) CCC( = O) CC( = O) CCC2 = CC( = C (C = C2) OC3C (C (C (C (O3) C( = O) O) O) O) O) OC) O	PubChem CID 24968346	0.639
Curcumin sulfate (20)	COC1 = C (C = CC( = C1) C = CC( = O) CC( = O) C = CC2 = CC( = C (C = C2) OS( = O)( = O) O) OC) O	PubChem CID 66645351	0.430
Curcumin glucuronide (21)	COC1 = C (C = CC( = C1) C = CC( = O) CC( = O) C = CC2 = CC( = C (C = C2) OC3C (C (C (C (O3) C( = O) O) O) O) O) OC) O	PubChem CID 71315012	0.557

*The score above shows the Pa (Probability active) value, whereas the Pi (Probability inactive) score is not displayed because it has a value <0.1

There are 20 curcumin derivatives collected that have the potential as “p53 enhancers,” with five derivatives of which are more potential than curcumin, such as hexahydrocurcumin compounds, which have a Pa score of 0.837, then tetrahydrocurcumin with Pa 0.752, curcumin diglucoside (3) with Pa 0.728, octahydrocurcumin (17) with a Pa score of 0.720, and demethoxycurcumin (14) with Pa 0.693, while curcumin (1) has a Pa score of 0.671. When the Pa score approaches 1, bioactivity is stronger [41]. The differences among the functional groups of each curcumin derivative result in differences in biological activity. Curcumin has methoxy and hydroxy substituents with the same pattern on both benzene chains, whereas the aliphatic heptadiene structure contains two keto groups. Hexahydrocurcumin (16) also has methoxy and hydroxy substituents with the same pattern on both benzene chains, but in the heptadiene structure, it undergoes dehydrogenation so that it only contains one hydroxy and one keto functional group each. Thus, hexahydrocurcumin (16) is more hydrophilic than curcumin (1), and its geometric structure of curcumin (1) tends to be more planar than the geometric structure of hexahydrocurcumin (1), which forms an angle in the middle of the alkyl chain [45]. On the other hand, tetrahydrocurcumin (18), with a Pa score still higher than curcumin (1) also undergoes dehydrogenation of alkyl heptadiene, but still with 2 keto groups like curcumin (1) so that it is more hydrophobic. However, it is necessary to explore the physicochemical properties that play an important role in providing value as a “p53 enhancer” based on the equation formed from all ligands involved in the QSAR analysis [46].

### Analysis of physicochemical properties that play a role in producing effects as “p53 enhancers”

Furthermore, the “p53 enhancer” effect of curcumin and its 20 derivatives was examined for its physicochemical characteristics. The descriptor parameters used refer to the report of Frengki *et al*. [47], which used several descriptors representing 3 hydrophobic, electronic, and steric parameters. The descriptors used include lipophilicity parameters such as tpsa, logP, and logS; electronic parameters such as am1_homo and am1_dipole; and steric parameters such as molar refractivity (mr) and glob [48]. Table 2 shows the physicochemical data of 7 curcumin descriptors and 20 derivatives.

**Table 2:** Physicochemical data of 7 descriptors of curcumin and its 20 derivatives.

Compounds	Qsar_pred	am1_dipole	am1_homo	tpsa	logS	mr 2D	glob	logP (o/w)
Curcumin (1)	0.671	3.55	−8.60	93.06	−4.06	10.13	0.19	3.72
Bisdemethoxycurcumin (2)	0.646	6.43	−9.06	74.60	−3.96	8.86	0.20	3.74
Curcumin diglucoside (3)	0.728	2.15	−9.06	254.52	−3.57	16.73	0.33	−0.53
Dimethylcurcumin (4)	0.631	2.03	−8.48	74.22	−4.85	11.15	0.36	4.52
Didemethylcurcumin (5)	0.653	3.85	−8.63	115.06	−3.23	9.12	0.15	3.19
Dimethoxycurcumin (6)	0.591	4.00	−8.84	71.06	−4.88	11.15	0.42	3.75
α-curcumene (7)	0.532	0.17	−9.08	0.00	−5.22	6.76	0.13	5.72
β-curcumene (8)	0.557	0.17	−8.89	0.00	−4.69	6.75	0.15	5.73
Monodemethylcurcumin (9)	0.671	3.33	−8.72	104.06	−3.65	9.63	0.11	3.45
Pentagamavunon-1 (10)	0.644	2.15	−8.66	57.53	−4.32	10.34	0.07	5.09
Daiarylpentadienone (11)	0.61	3.40	−8.76	37.30	−4.73	7.72	0.04	4.40
(2-hydroxy-4-[(1E,6E)-7- (4-hydroxy-3- methoxyphenyl)-3,5- dioxo-1,6- heptadien-1-yl]-2 methoxyphenylester) (12)	0.644	5.08	−8.89	119.36	−6.17	13.34	0.13	5.06
Dibenzoylmethane (13)	0.61	3.91	−9.86	34.14	−3.67	6.83	0.07	3.06
Demethoxycurcumin (14)	0.693	4.49	−8.66	83.83	−4.01	9.50	0.51	3.73
Dihydrocurcumin (15)	0.6	3.58	−8.76	133.52	−3.09	10.38	0.33	2.73
Hexahydrocurcumin (16)	**0.837**	**2.92**	**−8.59**	**96.22**	**−2.72**	**10.28**	**0.17**	**3.01**
Octahydrocurcumin (17)	0.72	1.83	−8.59	99.38	−2.83	10.32	0.04	3.59
Tetrahydrocurcumin (18)	0.752	2.89	−8.70	105.20	−4.37	12.31	0.30	2.77
Tetrahydrocurcumin glucuronide (19)	0.639	1.81	−8.61	189.28	−2.91	13.60	0.16	0.47
Curcumin sulfate (20)	0.43	4.93	−8.76	136.43	−4.60	11.23	0.21	2.82
Curcuminglucuronide (21)	0.557	4.14	−8.83	189.28	−4.03	13.50	0.22	1.40
Average	0.64 ± 0.08^a^	3.18 ± 1.54^a^	−8.81 ± 0.29^a^	98.48 ± 61.4^b^	−4.07 ± 0.87^c^	10.46 ± 2.51^d^	0.20 ± 0.13^a^	3.40 ± 1.56^a^

^a,b,c,d^Different superscripts in the same column indicate significant differences (p < 0.05). The bold values show the compound with activity as the strongest "p53 enhancer"

The QSAR equation model was developed using SPSS software with the MLR method. A leave-one-out cross-validation with q2 parameters was performed. The q2 value that meets the requirements is ≥0.5. The selected QSAR model had the highest q2 value. This MLR analysis of all descriptors except am1_homo as the equation axis component (x) and the activity of “p53 enhancer” as the axis value (y) is as follows.

Y = 0.588 + 0.011 (am1_dipole) + 0.013 (logP) + 0.107 (logS), 0.002 (tpsa), 0.013 (glob) + 0.061 (mr)

With R = 0.752, R Square = 0.565, Standard error = 0.067

The QSAR technique using the Way2Drugs website can automatically predict the potential biological activity of curcumin and its derivatives as “Pa enhancers” through the Pa score, whereas the cause of the high Pa score value is determined by the QSAR technique through descriptor analysis using SPSS.

There are 3 descriptors that showed a significant role (p < 0.05) in the results of further statistical analysis using the “Duncan” method. These descriptors are molar refractivity (mr 2D), which represents steric parameters; solubility (logS); and tpsa, which represents hydrophobicity parameters. Molar refractivity is used to express the steric properties of compounds that can affect drug interactions with receptors. The greater the molar refractivity result, the greater the steric properties obtained, but the drug interaction with the receptor is not good. Conversely, a lower molar refractivity value indicates greater hydrophobic properties; this hydrophobic property makes it easier for the compound to bind to the receptor [49]. High solubility in fat is less well absorbed than drugs that are soluble in water, especially if the drug is enteral [50]. The tpsa is used to analyze the ability of drugs to enter cells [51]. The ability of a molecule to interact with a receptor is greatly influenced by the ability of the compound to match its conformation in the receptor cavity [52]. Based on the regression equation (1) above, it shows that the more positive the value of the mr and logS descriptors of a ligand, the more its pharmacological effect will increase, thus increasing the hydrophilicity of curcumin derivatives will increase the effect of the “p53 enhancer.” Conversely, in the tpsa descriptor, the more positive the value of a ligand is, the more its pharmacological effect will decrease. Table 2 shows that the values of the mr 2D and logS descriptors of hexahydrocurcumin (16) are −2.72 and 10.28, respectively, compared with the mr and logS descriptors of curcumin (1) −4.06 and 10.13, respectively, indicating that hexahydrocurcumin (16) has a tendency to be more hydrophilic (+). These two descriptors (mr 2D and logS) are responsible for the higher bioactivity of hexahydrocurcumin (16**)** higher than curcumin (1). The steric compatibility of hexahydrocurcumin (16) with the wild-type p53 receptor cavity and its better solubility compared with curcumin (1) is what increases its bioactivity as a “p53 enhancer.” The QSAR technique using the Way2Drugs website can automatically predict the potential biological activity of curcumin and its derivatives as “Pa enhancers” through Pa scores, whereas the cause of the high Pa score value is determined by the QSAR technique through descriptor analysis using SPSS. Furthermore, an analysis of the interaction strength (affinity) of curcumin and its derivatives was performed using the molecular docking method.

### Molecular docking curcumin and its derivatives as a “p53 enhancer” and as “p53 mutant R273H reactivator”

The potential as a “p53 enhancer” of the QSAR analysis results of curcumin compounds and 20 of its derivatives was confirmed by the strength of its affinity through the molecular docking method against wild-type p53 and mutant p53 proteins. Humanized xenopus mdm2 (4lwu) and p53 mutant (4ibs) proteins were selected as receptors based on models reported by Zhang *et al*. [53] and Eldar *et al*. [37]. The affinity parameters observed were hydrogen bonds at amino acid Leu 50 (4lwu) and hydrogen bonds at amino acid His 273 (4ibs). Each of these hydrogen bonds was identified as an important factor in the mdm2 inhibitory effect and the p53 mutant R273H reactivation effect. Another parameter observed is the free energy (ΔGbinding), which is released spontaneously due to the formation of the ligand-receptor complex; the greater the ΔGbinding released indicates a stronger interaction [52, 54].

**Figure 1:**
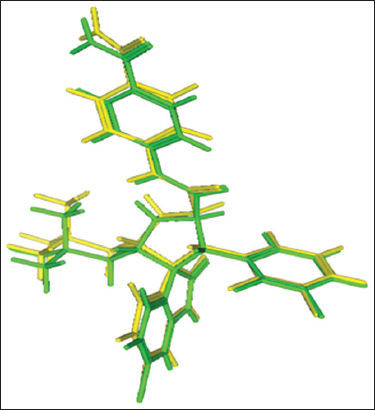
Root means square deviation of native ligand 2ou (yellow) versus copy ligand 2ou (green) with a score of 0.497 Å.

Before testing, the docking method was first validated against the native ligand 2ou humanized xenopus mdm2 receptor (4lwu), and a RMSD value of 0.666 Å was obtained, so that the docking method met the validity requirements and could be continued for docking the test compound. Figure 1 shows the RMSD value of the native ligand 2ou against its copy.

In contrast, the p53 mutant R273H (4 ibs) receptor did not validate the method because it does not have a native ligand. Thus, docking was performed directly against the “site finder” selected directly by the MOE system. The molecular docking results in Table 3 show the ∆Gbinding and Hydrogen bonds of curcumin and its 20 derivatives to the humanized xenopus mdm2 receptor and p53 mutant R273H as follows.

**Table 3:** ∆Gbinding and hydrogen bonds of curcumin and 20 of its derivatives on humanized xenopus mdm2 (4lwu) and p53 mutant R273H (4ibs) receptors.

Compounds	Humanized xenopus mdm2 (4lwu)		Reseptor p53 mutant R273H (4ibs)	
	
	∆G*_binding_* (kcal/mol) ± Standard	Hydrogen Bond	∆G*_binding_* (kcal/mol) ± Standard	Hydrogen Bond
Curcumin (1)	**−8.62 ± 0.32**	**Leu 50, Glu 65**	**−9.61 ± 0.12**	**His 273, Arg 248 (2), Asp 281, Glu 285**
Bisdemethoxycurcumin (2)	−9.53 ± 0.56	Tyr = 51, Gln = 55	**−10.08 ± 0.98**	**Ser 241 (2), His 273 (2), Arg 248 (2)**
Curcumin diglucoside (3)	−11.55 ± 0.87	Tyr 96 (2), Lys 47	−12.92 ± 1.09	**Gln 136, Leu 137, His 273 (2), Asp 281, Glu 285 (2), Lys 139 (3), Arg 248**
Dimethylcurcumin (4)	−7.77 ± 0.19	Tyr 96 (2)	−9.37 ± 0.95	Glu, 285; Lys, 132; Arh, 248
Didemethylcurcumin (5)	**−7.93 ± 0.21**	**Leu 50 (2), Gln 55**	**−13.17 ± 2.02**	**His 273 (2), Asp 281, Glu 285, Lys 132, Arg 248 (2), Ala 276**
Dimethoxycurcumin (6)	−8.57 ± 0.23	Gln 55	−12.33 ± 0.78	Lys 132, Ser 240, Arg 248 (2)
α-curcumene (7)	−7.21 ± 0.12	-	−7.65 ± 0.17	-
β-curcumene (8)	−7.38 ± 0.18	-	−6.77 ± 0.28	-
Monodemethylcurcumin (9)	**−6.86 ± 0.09**	**Leu 50, Glu 65**	**−11.87 ± 0.88**	**His 273 (2), Glu 285, Lys 132, Arg 248 (2)**
Pentagamavunon-1 (10)	−9.24 ± 0.31	Tyr 96 (2)	−9.83 ± 1.12	Ser 241 (2)
Daiarylpentadienone (11)	−8.28 ± 0.32	-	−7.79 ± 0.56	Asp 281 and Arg 248 (2)
(2-hydroxy-4-[(1E,6E)-7- (4-hydroxy-3- methoxyphenyl)-3,5-dioxo- 1,6-heptadien-1-yl]- 2-methoxyphenylester) (12)	−8.87 ± 0.23	**Leu 50, Lys 47 (2)**	−9.70 ± 0.34	Glu, 271; Asp, 281; Lys, 132; Lys, 164; Arg, 248
Dibenzoylmethane (13)	−7.17 ± 0.14	-	−9.64 ± 1.27	Arg 248 (2)
Demethoxycurcumin (14)	−8.25 ± 0.24	Val 89, Gln 55	**−11.45 ± 0.57**	**His 273 (2), Val 274, Ser 241, and Arg 248 (2)**
Dihydrocurcumin (15)	−8.78 ± 0.45	Tyr 63 (2) Glu 65, Lys 6, His 92, and Tyr 9	**−11.87 ± 0.87**	**His 273 (2), Asp 281 (2), Glu 285, Arg 248 (2)**
Hexahydrocurcumin (16)	**−7.67 ± 0.27**	**Leu 50**	−12.07 ± 0.77	Glu 285; Lys 132; Arg 248
Octahydrocurcumin (17)	−8.95 ± 0.67	Glu 65	−13.06 ± 0.98	Asp 281, Glu 285, Arg 248, Ala 276
Tetrahydrocurcumin (18)	−7.73 ± 0.15	Gln 55	−10.50 ± 1.22	Lys 164, Arg 248
Tetrahydrocurcumin glucuronide (19)	**−12.18 ± 1.22**	**Leu 50, Tyr 51, Gln 55 (2)**	−13.38 ± 1.31	Ser 241 (2), Lys 132 (2), Arg 248 (2) Ala 276
Curcumin sulfate (20)	−7.94 ± 0.23	Gln 55 and Tyr 96 (2)	−12.87 ± 1.65	Ser 241 (3), Lys 132, Arg 248 (4), and Asn 239
Curcumin glucuronide (21)	−10.48 ± 1.12	Gln 65 and Lys 66 (2)	−12.04 ± 1.89	Glu 271
2ou (inhibitor mdm2)	−12.23 ± 1.78	**Leu, 50; His, 92**	-	-

The bold values showed the test compounds to form hydrogen bonds with Leu 50 in MDM2 and or His 273 receptors in p53 Mutant receptors

**Figure 2:**
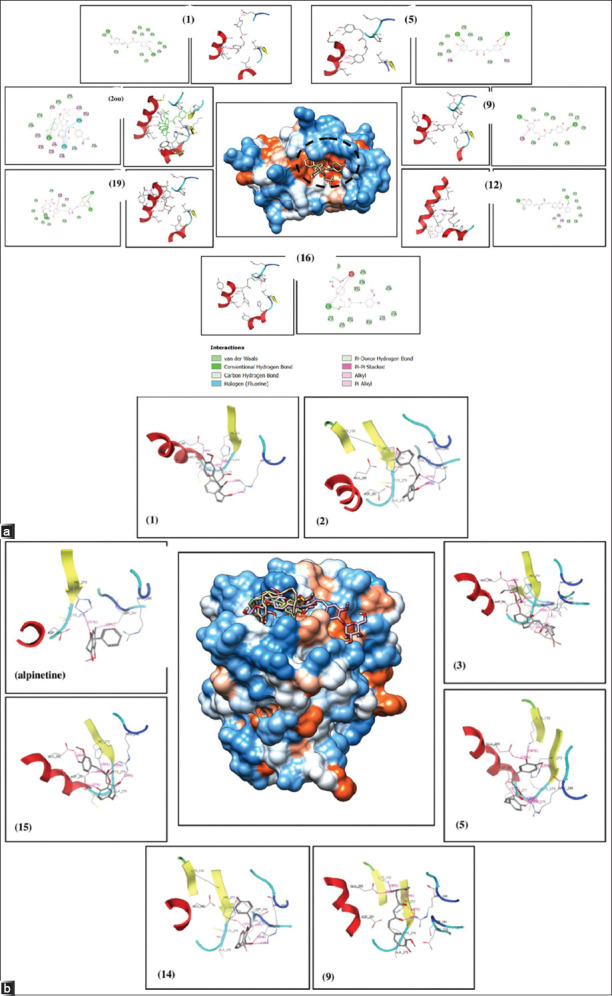
(a) 2D and 3D visualization of curcumin (1), didemethylcurcumin (5), monodemethylcurcumin (9), (2-hydroxy-4-[(1E,6E)-7-(4-hydroxy-3-methoxyphenyl)-3,5-dioxo-1,6-heptadien-1-yl]-2-methoxyphenylester (12), hexahydrocurcumin (16), tetrahydrocurcumin glucuronide (19), and native ligand (2ou) against mdm2 receptor (pdb id.4lwu), which has hydrogen bond with leucine 50. (b) 2D and 3D visualization of curcumin (1), bisdemethoxycurcumin (2), didemethylcurcumin (5), monodemethylcurcumin (9), demethoxycurcumin (14), hexahydrocurcumin (15), and alpinetine control against the p53 mutant R273H receptor (pdb id. 4ibs), which has a hydrogen bond with Histidine 273. *The dotted circle line indicates the binding site.

The results of molecular docking showed that only six compounds interacted with the mdm2 receptor, forming 1 hydrogen bond as the native ligand 2ou, namely, curcumin (1), didemethylcurcumin (5), monodemethylcurcumin (9), and (2-hydroxy-4-[(1E,6E)-7-(4-hydroxy-3-methoxyphenyl)-3,5-dioxo-1,6-heptadien-1-yl]. -2-methoxyphenylester, hexahydrocurcumin (12), and tetrahydrocurcumin glucuronide (19). Meanwhile, molecular docking on the p53 mutant R273H receptor showed seven compounds that interacted with the target, forming 1 hydrogen bond with His 273, as reported by Malami *et al*. [22]. The seven compounds were curcumin (1); bisdemethoxycurcumin (2); didemethylcurcumin (5); monodemethylcurcumin (9); demethoxycurcumin (14); and hexahydrocurcumin (15). As a control compound, alpinetin also showed hydrogen bonds with His 273. Figures 2a and b show the 2D and 3D visualization of the molecular docking results for the target proteins mdm2 and p53.

The molecular docking of curcumin (1) and 20 of its derivatives against the humanized xenopus mdm2 receptor (4lwu) produced a lower ∆Gbinding value than the 2ou control as a native ligand (Table 3), but curcumin (1) and 5 of its derivatives have interactions with the amino acid Leucine 50 on the receptor as the native ligand 2ou. Curcumin (1) releases energy with ∆Gbinding −8.622 (kcal/mol) by forming 2 hydrogen bonds (Leu 50, Glu 65), didemethylcurcumin (5) releases energy with ∆Gbinding −7.931 (kcal/mol) by forming 3 hydrogen bonds (Leu 50 [2], Gln 55), monodemethylcurcumin (9) releases energy with ∆Gbinding −6.83 (kcal/mol) by forming 2 hydrogen bonds (Leu 50, Gln 55), (2-hydroxy-4-[(1E,6E)-7-(4-hydroxy-3-methoxyphenyl)-3,5-dioxo-1,6-heptadien-1-yl] -2-methoxyphenylester) (12) releases energy with ∆Gbinding −8.874 (kcal/mol) by forming 3 hydrogen bonds (Leu 50, Lys 47 [2]), hexahydrocurcumin (16) releases energy with ∆Gbind-ing −7.674 (kcal/mol) by forming 1 hydrogen bond (Leu 50), and tetrahydrocurcumin glucuronide (19) releases energy with ∆Gbinding −12.182 (kcal/mol) almost equivalent to the native ligand 2ou by forming 4 hydrogen bonds (Leu 50, Tyr 51, Gln 55 [2]). The other 15 curcumin derivatives did not form the same hydrogen bonds as the native ligand 2ou. The Pa score as a “p53 enhancer” does not correlate with the affinity value (∆Gbinding), which makes sense because the ligand-receptor hydrogen bond is not the sole factor that determines the pharmacological effect, but hydrophobic bonds and stereochemical effects also play a significant role in pharmacological effects [55–57].

### MD of curcumin and its derivatives as “p53 mutant R273H reactivator”

**Figure 3:**
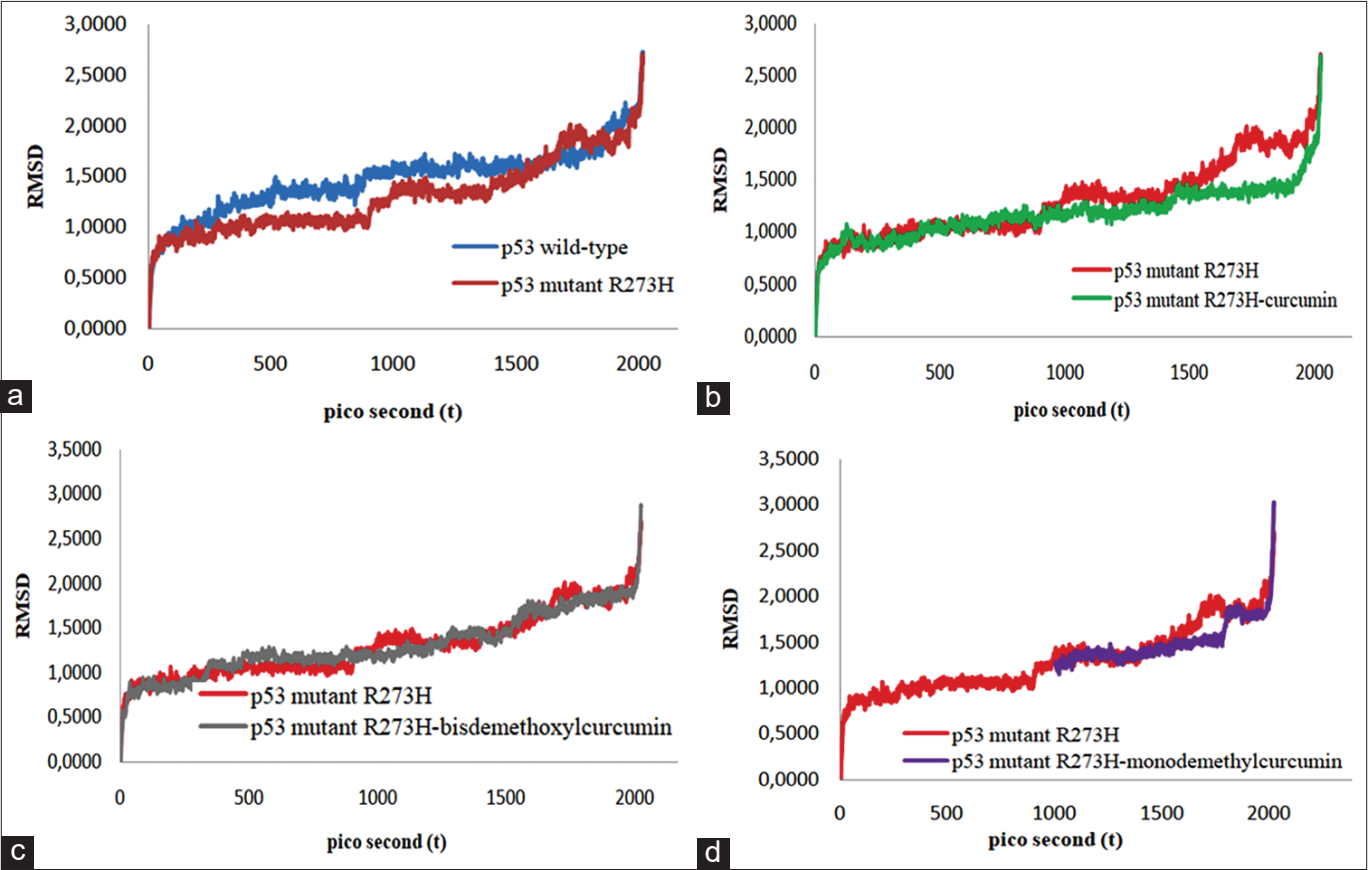
Comparison of RMSD and fluctuation patterns of (a) p53 wild-type vs p53 mutant R273H, (b) p53 mutant R273H vs p53 mutant R273H-curcumin(1), (c) p53 mutant R273H vs p53 mutant R273H-bisdemethoxycurcumin(2), and (d) p53 mutant R273H vs p53 mutant R273H-monodemethylcurcumin(9).

The interaction that occurs in the docking results shows the condition of the enzyme in a rigid state; therefore, it needs to be evaluated in a hydrated molecular state using the MD simulation method. The deviation of the molecular conformation during the MD simulation is observed in the RMSD value. RMSD is a measure that is often used in the analysis of 3D molecular geometry to compare changes or shifts in molecular conformation. However, the fluctuation pattern of the amino acids in the protein determines the RMSD value [58]. The simulation results show the fluctuation of wild-type p53 residues, p53 mutant R273H, and p53 mutant R273H, which form a complex with curcumin (1) and curcumin derivatives during the 2ns simulation process. Observations were made on a nanosecond time scale (10–9) because on this time scale, the movement of the relatively rigid backbone (rigid body motion) can be observed, including the movement of the α-helical strands and the movement of the enzyme domain. In Figure 3, the fluctuation pattern of the p53 mutant R273H tended to be unstable compared to p53 wild-type. Curcumin (1), bisdemethoxycurcumin (2), and monodemethylcurcumin (9) tend to form dynamic patterns similar to those of p53 wild type when interacting with p53 mutant R273H.

The complexes of p53-mutant R273H-curcumin (1), p53-mutant R273H-bisdemethoxycurcumin (2), and p53-mutant R273H-monodemethylcurcumin (9) appear more stable than p53-mutant R273H when the MD simulation was run for 2 ns. Thus, curcumin (1), bisdemethoxycurcumin (2), and monodemethylcurcumin (9) are estimated to be able to suppress the fluctuation of p53 mutant R273H to be more stable and have the potential to become a reactivator compound of p53 mutant R273H so that it has the potential to restore its function as wild-type p53.

The interaction pattern and hydrogen bonding between curcumin (1) and several of its derivatives with amino acid histidine 273 and several amino acids that are the site binding of the p53 mutant enzyme during the dynamics simulation did not experience significant differences. The interaction of curcumin (1) and bismethoxycurcumin (2) through hydrogen bonds to the amino acid histidine 273 of the mutant p53 enzyme still occurred at observation times of 0 pc, 500 pc, 1000 pc, 1500 pc, and 2000 pc. While monodemethylcurcumin (9) only showed a difference in not interacting with histidine 273 of the mutant p53 during observation at 500 pc (Table 4). Based on these 3 ligands, it can be interpreted that curcumin (1) and some of its derivatives can reactivate the p53 mutant R273H to function again as wild-type p53.

**Table 4:** Contact residues of curcumin (1), bisdemethoxy curcumin (2), and monodemethyl curcumin (9) on p53 mutant R273H during molecular dynamics simulations at 310°K.

Dymanic’s times	p53-mutant R273H-curcumin (1)	p53-mutant R273H-bisdemethoxycurcumin (2)	p53 mutant R273H-monodemethylcurcumin (9)
0	His 273, Glu 285; Arg 248; Asp 281; Asn 239; Ala 276; Cys 275; Cys 277	His 273, Glu 285; Arg 248, Cys 275; Cys 277; Ala 276; Asn 239	His 273, Glu 285; Arg 248; Cys 275; Ala 276; Asp 281; Cys 277
500 ps	His 273, Glu 285; Lys 132; Arg 248; Ala 276; Ser 241	His 273, Glu 285; Lys 132; Arg 248; Ser 241; Ala 276	Glu, 285; Arg, 248; Ala, 276; Asp, 281
1000 ps	His 273, Glu 285; Lys 132; Arg 248; Ala 276; Ser 241; Asp 281	His 273, Glu 285, Lys 132, Arg 248, Asp 281, Ser 241, Ala 276	His 273, Glu 285; Arg 248; Ala 276; Asp 281; Cys 277
1500 ps	His 273, Glu 285, Arg 248, Ala 276, Ser 241, and Asp 28	His 273, Glu 285, Arg 248, Asp 281, Ser 241, Ala 276	His 273, Glu 285; Arg 248; Ala 276; Asp 281; Cys 277
2000 ps	His 273, Glu 285; Arg 248; Ala 276; Ser 241; Asp 28; Pro 250	His 273, Glu 285, Arg 248, Asp 281, Ser 241, Ala 276	His 273, Glu 285; Arg 248; Ala 276; Asp 281; Cys 277

For the development of candidate drugs, especially several curcumin derivatives that are considered more promising, it is necessary to consider the pharmacokinetic and toxicity properties of the candidate drugs, especially when given orally.

### Profile of the pharmacokinetics and toxicity of curcumin and its 20 derivatives

In drug development, early prediction of pharmacokinetic and toxic properties is crucial to avoid costly and unnecessary failures. The adsorption, distribution, metabolism, excretion, and toxicity of the test compounds were predicted through the pkCSM and tox_ii web servers. Table 5 shows the pharmacokinetic-toxicity profile of curcumin and its 20 derivatives based on the analysis of the two web servers.

**Table 5:** Pharmacokinetic-toxicity profile of curcumin and its 20 derivatives based on pkcsm and protox_ii analyses.

Compounds	Absorption		Distribution		Excretion	Metabolism							Toxicity	
				
	HIA (%)	Caco-2 Log (10^-6^ cm/s)	PPB (%)	BBB	CLtot (log.mL/min/kg)	2D6 substrat	3A4 substrate	1A2 inhibition	C19 inhibitor	2C9 inhibitor	2D6 inhibitor	3A4 inhibitor	LD_50_ (g/kg BB)	Level of toxicity
(1)	81.719	0.556	0.103	−0.677	0.014	No	Yes	No	Yes	Yes	No	Yes	2 g	IV
(2)	91.159	0.957	0.045	−0.089	−0.008	No	Yes	Yes	Yes	Yes	No	Yes	2.56	V
(3)	23.912	−1.15	0.376	−0.638	0.761	No	Yes	No	No	No	No	No	4	V
(4)	91.431	1.040	0.038	−0.68	0.214	No	Yes	No	Yes	Yes	No	Yes	4	V
(5)	73.185	−0.485	0.172	−0.999	−0.076	No	Yes	No	No	No	No	No	2.56	V
(6)	95.408	1.13	0	−0.783	0.13	No	Yes	Yes	Yes	Yes	No	Yes	2	IV
(7)	93.29	1.537	0.015	0.593	1.511	No	Yes	Yes	No	No	No	No	2	IV
(8)	95.232	1.419	0.231	0.788	1.44	No	No	No	No	No	No	No	3.65	V
(9)	75.291	−0.463	0.039	−1.158	−0.045	No	Yes	No	Yes	No	No	No	2	IV
(10)	89.664	0.971	0	−0.217	0.339	No	Yes	Yes	Yes	Yes	No	Yes	4.4	V
(11)	94.18	1.613	0	0.008	0.113	No	Yes	Yes	Yes	No	No	No	1	IV
(12)	86.587	0.567	0	−1.063	0.009	No	Yes	No	Yes	Yes	No	Yes	3.45	V
(13)	96.744	1.318	0.059	0.323	0.229	No	Yes	Yes	Yes	No	No	No	1	IV
(14)	91.393	1.023	0	−0.337	0.026	No	Yes	Yes	Yes	Yes	No	Yes	2	IV
(15)	72.23	−0.842	0.047	−1.498	0.086	No	No	No	No	No	No	No	3	V
(16)	75.258	0.389	0.156	−1.055	0.320	No	Yes	No	No	No	No	No	0.25	III
(17)	72.382	−0.229	0.018	−1.02	0.682	No	Yes	Yes	Yes	Yes	No	No	2 g	IV
(18)	93.588	1.032	0	−1.257	1.428	No	Yes	No	Yes	Yes	No	Yes	3.038	V
(19)	34.629	−0.488	0.288	−1.907	0.845	No	No	No	No	No	No	No	4	V
(20)	60.902	0.793	0.007	−1.365	0.047	No	No	No	No	No	No	No	4	V
(21)	25.688	−0.582	0.145	−1.52	−0.328	No	No	No	No	No	No	No	5	V
2ou	96.067	1.198	0	−0.99	0.315	No	Yes	No	Yes	No	No	Yes	5	IV

LD_50_ = Lethal dose 50%, HIA = Human intestinal absorption, PPB = Plasma protein binding, BBB = Blood-brain barrier

The evaluation of the pharmacological effects of each bioactive compound is usually followed by an observation of its toxicity potential. Prediction of the toxicity potential of curcumin and its 20 derivatives using the LD_50_ mol_/_kg parameter and the toxicity level. LD_50_ is a single treatment dose or from several treatments in a short time that can cause 50% death in a group of experimental animals [59]. The toxicity level of curcumin and its derivatives is at level IV-V except for hexahydrocurcumin (level III) with an LD_50_ of 0.25 g/kg BW; thus, curcumin (1) and its derivatives are safe for oral consumption.

The observed absorption profile predicts the HIA value and Caco-2 cell permease. HIA shows the degree of absorption of active substances in the human intestine. If a compound’s percentage HIA value falls between 70% and 100%, it is considered well absorbed, sufficient in the range of 20%–70%, and poor in the range of 0%–20% [60]. Curcumin and its derivatives have an HIA value of >70%, so they experience good absorption in the digestive tract, except for curcumin derivatives with large molecules such as curcumin diglucoside (3), tetrahydrocurcumin glucuronide (19), and curcuminglucuronide (21).

The observed drug distribution parameters are PPB and BBB. PPB >90% is predicted to mean that the drug is strongly bound to plasma protein; conversely, if PPB <90%, it is predicted that the drug is weakly bound to plasma protein so that it can be distributed well to its target [44]. Based on Table 5, curcumin and its 14 derivatives show strong binding to plasma proteins; only curcumin diglucoside (3), didemethylcurcumin (5), β-curcumene (8), hexahydrocurcumin (16), tetrahydrocurcumin glucuronide (19), and curcuminglucuronide (21) show strong binding to plasma proteins, so it is suspected that all five are distributed limitedly to body tissue. The other distribution parameters of BBB indicate the ability of a drug to reach brain tissue. In the pkCSM predictive model, BBB values are classified into two categories, namely, values >0.3 are considered easy to pass through the BBB, whereas molecules and values <−1 are not well distributed to the brain. Based on Table 5, it shows that only α-curcumene (7), β-curcumene (8), and dibenzoylmethane (13) have the potential to penetrate the BBB, with BBB values of 0.593, 0.788, and 0.323. In addition, it is predicted that it will be less able to penetrate the BBB.

The observed metabolic profiles include CYP substrates and CYP inhibitors. CYP enzymes are a superfamily of isoenzymes that play important roles in drug elimination through metabolic biotransformation [61]. CYP450 has five main isoforms, namely CYP1A, CYP2C19, CYP2C9, CYP2D6, and CYP3A4 [44]. Identification of interactions through these cytochrome enzymes needs to be monitored for if drugs are given in combination because it has an impact on pharmacokinetic drug interactions that cause pharmacological effects to weaken because drug clearance increases, or conversely, toxic effects and unwanted drug reactions increase due to lower clearance and drug accumulation [62]. Among the curcumin derivatives, the compounds curcumin diglucoside (3), didemethylcurcumin (5), β-curcumene (8), dihydrocurcumin (15), hexahydrocurcumin (16), tetrahydrocurcumin glucuronide (19), curcumin sulfate (20), and curcumin glucuronide (21) are substrates for CYP enzymes but are not inhibitors for other CYPs. Thus, these eight curcumin derivatives are safer when used along with other drugs.

The excretion profiles of the liver, biliary gland, and kidneys are observed through the total excretion parameter value (CLtot) [44]. A compound excretion is said to be good if its molecular weight is small and hydrophilic. Conversely, if the compound has a high molecular weight and is hydrophobic, the elimination process takes a long time and is at risk of toxicity due to accumulation in the body if given repeatedly [63]. Based on Table 5, curcumin and the majority of its derivatives have low total clearance (<1), so they are predicted to last a long time in the body. Only α-curcumene (7) and tetrahydrocurcumin (18) had a total clearance of >1 (1511 and 1428, respectively). Both compounds are predicted to be eliminated quickly from the body [64].

**Figure 4:**
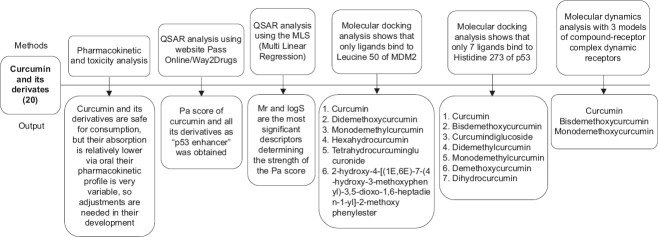
Overview of the results of quantitative structure-activity relationship analysis, docking, and molecular dynamics, as well as the pharmacokinetic and toxicity profiles of the findings.

However, the use of curcumin and its derivatives orally absorbed in the digestive tract is relatively unsatisfactory. Thus, various modifications of curcumin formulas are made to enhance their absorption. The use of curcumin nanoparticle preparations and its derivatives is a proven option, but it requires quite expensive costs [65, 66]. On the other hand, experimental data on curcumin derivatives are still very limited compared with curcumin that is already in the clinical trial stage. Therefore, it is very important to conduct research on curcumin, including its administration in nanoparticle preparations, to overcome its low absorption if given orally. The analysis of the relationship between quantitative structures, docking, and molecular dynamics, as well as pharmacokinetic profiles and toxicity of the findings are presented in Figure 4.

## CONCLUSION

This study demonstrated the potential of curcumin and its derivatives to function as p53 enhancers and reactivators of the p53 mutant R273H. QSAR analysis identified hexahydrocurcumin (Pa: 0.837) and tetrahydrocurcumin (Pa: 0.752) as the most potent p53 enhancers, with their steric and hydrophobic properties contributing significantly to their bioactivity. Molecular docking confirmed the strong binding affinities of curcumin derivatives to key residues of wild-type p53 and mutant p53R 273H, whereas MD simulations highlighted the stabilization of mutant p53 R273H by curcumin, bisdemethoxycurcumin, and monodemethylcurcumin. Pharmacokinetic predictions revealed favorable absorption and low toxicity profiles for most derivatives, with some compounds demonstrating promising drug-like properties.

The strength of this study lies in its comprehensive *in silico* approach, combining QSAR modeling, molecular docking, MD, and pharmacokinetics to provide a detailed evaluation of curcumin derivatives. The findings offer valuable insights into the dual roles of these compounds in enhancing wild-type p53 expression and reactivating mutant p53, presenting a strong basis for further exploration of their therapeutic potential.

However, this study has limitations. *In silico* analysis lacks experimental validation *in vitro* and *in vivo*, which are necessary to confirm the predicted bioactivity and pharmacokinetic properties. In addition, although several derivatives showed strong potential, their exact mechanisms of action and specificity to ward p53 remain to be elucidated. The present study also did not assess potential off-target effects or interactions with other biomolecules that may influence their therapeutic application.

Future research should focus on experimental validation of these findings, including cell-based assays and animal models, to establish the efficacy and safety of curcumin derivatives. Investigating their bioavailability, metabolic pathways, and potential synergy with existing anticancer agents would further strengthen their clinical relevance. In addition, structural optimization of promising derivatives can enhance their pharmacological profiles and broaden their therapeutic applications.

## AUTHORS’ CONTRIBUTIONS

SIN: Conceived and designed the *in silico* study. FF and SIN: Performed the molecular docking and molecular dynamics. RKK, FF, and MH: Performed the data analysis. RR: Performed the data analysis and drafted and revised the manuscript. All authors have read and approved the final manuscript.
